# Methyl jasmonate leads to necrosis and apoptosis in hepatocellular carcinoma cells via inhibition of glycolysis and represses tumor growth in mice

**DOI:** 10.18632/oncotarget.17469

**Published:** 2017-04-27

**Authors:** Jingjing Li, Kan Chen, Fan Wang, Weiqi Dai, Sainan Li, Jiao Feng, Liwei Wu, Tong Liu, Shizan Xu, Yujing Xia, Jie Lu, Yingqun Zhou, Ling Xu, Chuanyong Guo

**Affiliations:** ^1^ Department of Gastroenterology, Shanghai Tenth People's Hospital, Tongji University School of Medicine, Shanghai 200072, China; ^2^ Department of Gastroenterology, Shanghai Tenth People's Hospital, School of Clinical Medicine of Nanjing Medical University, Shanghai 200072, China; ^3^ Department of Gastroenterology, Shanghai Tongren Hospital, Shanghai Jiao Tong University School of Medicine, Shanghai 200336, China

**Keywords:** Warburg effect, hexokinase 2, hepatoma, methyl jasmonate, glycolysis

## Abstract

Methyl jasmonate has recently been found to have anti-cancer activity. Methyl jasmonate detached hexokinase 2 from a voltage dependent anion channel causing a reduction in mitochondrial transmembrane potential that led to the release of cytochrome C and apoptosis inducing factor resulting in intrinsic apoptosis. Blocked adenosine triphosphate synthesis caused by mitochondrial injury hampered oxidative phosphorylation and led to cell necrosis. The results were applied to the *in vivo* treatment of nude mice with a satisfactory effect. Collectively, our results suggest that methyl jasmonate may be an adjuvant therapy for liver tumors due to its mechanism in cancer cells compared to that in normal cells: The major function is to inhibit glycolysis instead of changing aerobic metabolism.

## INTRODUCTION

Hepatocellular carcinoma (HCC) is a malignant disease that threatens human health which results in disordered cell growth, proliferation, differentiation, and the apoptosis mechanism [1–3]. In 2000, Hanahan and Weinberg summarized six hallmarks of cancer cells: sustaining proliferative signaling, resisting cell death, inducing angiogenesis, enabling replicative immortality, activating invasion and metastasis and evading growth suppressors [4]. In the early 1930s, Otto Warburg made the seminal discovery known as the “Warburg effect”, and this was confirmed in a growing number of tumor types when fluorodeoxygucose positron emission tomography (FDG-PET) was used to detect glucose intake in tissue samples in 1990s [5]. Thus, the uncontrollability of gene mutation in tumor therapy is a dilemma that may be improved by interfering with energy metabolism.

The Warburg effect showed that tumor cells prefer glycolysis to oxidative phosphorylation (OXPHOS) in terms of glucose metabolism even in the presence of sufficient amounts of oxygen. This is because glycolysis results in the production of fatty acids, nucleic acids, proteins and membrane phospholipids that combine with ATP to meet the demand for tumor proliferation [5–7]. Hexokinase (HK) is not only the first enzyme in the glycolytic pathway but is the rate-limiting enzyme [8, 9]. Four HK isozymes have been identified and are known as HK1, 2, 3 and 4, of these enzymes, HK2, which is highly expressed in cancer, plays its role effectively by combining with the voltage dependent anion channel (VDAC) located on the mitochondrial outer membrane due to its hydrophobic N-terminal region [10–13].

Cancer cells are unlike normal cells in terms of metabolic behavior, and a myriad of new small molecular weight natural or synthetic compounds have been developed to accomplish the ultimate goal of killing tumor cells but not normal cells. Methyl jasmonate (MJ), is a plant stress hormone belonging to the jasmonate family, first isolated from the jasmine plant and is similar to the salicylate [2, 14]. The natural cyclopentanone lipid has similar function but not structure. However, unlike the side effects associated with salicylates, such as aspirin, MJ can arrest the cell cycle or cause cell death *in vitro* and *in vivo*, without affecting normal cells [15]. Previous research demonstrated the anti-cancer effects of MJ and its suggested mechanisms, in breast, colon, gastric, lung, myeloid leukemia, prostate, and sarcoma cancer cells [16–23]. In addition, the antinociceptive effects of MJ in experimental animals have important clinical significance in cancer treatment which were identified by Umukoro and colleagues [24]. Park showed that J7, a MJ derivative, enhanced apoptosis of hepatoma Hep3B and HepG2 cells by the generation of reactive oxygen species [25, 26]. However, the pharmacological mechanism was not identified. Goldin in 2008 showed that MJ binds to and detaches mitochondria-bound HK [27], and, HK2 is highly expressed in hepatoma. Therefore, MJ may function by affecting the combination of HK2 and VDAC1 which occurs in other types of hepatoma cells.

In the present study, we investigated the anti-cancer effects of MJ and its mechanism of action. For this purpose, we evaluated related markers of possible biological behavior including apoptosis, necrosis and mitochondrial injury at multiple levels.

## RESULTS

### Characterization of energy metabolism in HCC cells

**Figure 1:**
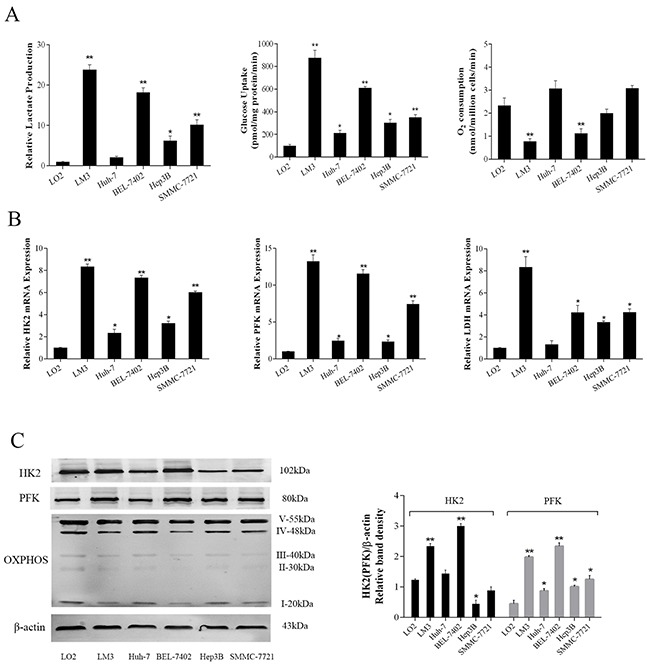
Metabolic features of HCC cells (**A**) Lactate production, glucose uptake and O_2_ consumption in normal liver cells (LO2) and HCC cells (LM3, Huh-7, BEL-7402, Hep3B and SMMC-7721) under aerobic conditions for 72 h. (**B**) The mRNA expression of HK2, PFK and LDH were determined by real-time PCR. (**C**) The protein levels of HK2, PFK and OXPHOS were detected by western blot. The ratios of HK2, PFK and β-actin were calculated by the Odyssey two-color infrared laser imaging system. The data are expressed as the mean±SD (n=3, *P<0.05 and **P<0.01 for HCC cells versus LO2 cells).

Malignant tumors often have an increased intake of glucose due to rapid proliferation [8]. In order to investigate the characteristics of energy metabolism in tumor cells, we preliminarily determined the levels of substrate consumption and mesostate production. As shown in Figure 1A, various HCC lines (LM3, BEL-7402, Hep3B, SMMC-7721) showed increased levels of lactate production and glucose uptake compared with normal liver cells (LO2), particularly LM3 and BEL-7402 (P<0.01). Associated with these results, O_2_ consumption in the LM3 and BEL-7402 cell lines (P=0.002, 0.005, respectively) was significantly reduced. These results demonstrated that energy metabolism in liver cancer cells was more active than that in healthy cells and glycolytic energy supply may increase compared with oxidative phosphorylation.

During glycolysis, glucose was decomposed to pyruvate by specific enzymes including HK2, PFK and LDH. The data (Figure 1B&1C) showed that the expression of related enzymes in cancer cells had heterogeneity compared to normal cells. The gene expression of HK2 and PFK in various cancer cell lines was higher than that of LO2, but their protein expression is lower except LM3 and BEL-7402. Marked differences in gene expression of LDH were seen in LM3, BEL-7402 and SMMC-7721, while no obvious difference was observed in Huh-7 (P=0.34). In addition, OXPHOS complexes that were used to oxidize nutrients, showed reduced expression in cell lines with increased glycolytic enzymes except Huh-7 [28]. These results clearly illustrate that glycolysis is the main power in tumor cell proliferation and oxidative phosphorylation played an important part in normal cells.

### Methyl jasmonate induced necrosis and caspase-independent apoptosis in HCC cells

**Figure 2:**
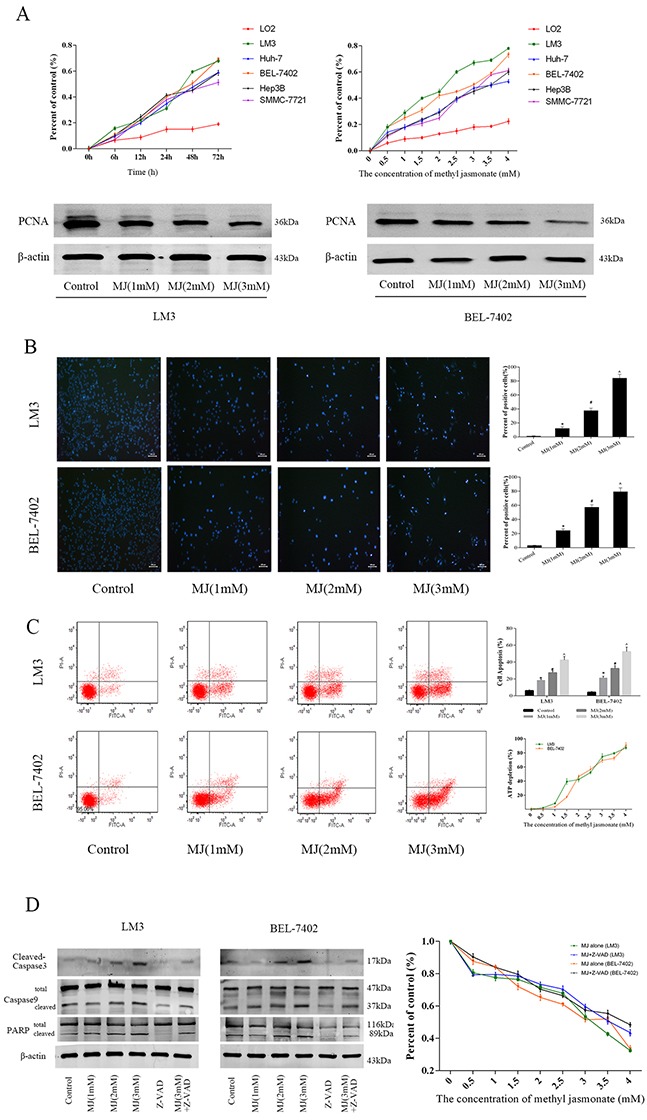
The effects of methyl jasmonate on HCC cells (**A**) Normal liver cells (LO2) and HCC cells (LM3, Huh-7, BEL-7402, Hep3B and SMMC-7721) were treated with MJ (0-4 mM) for 6–72 h. The CCK8 kit was used to monitor cell proliferation (n=3). The protein levels of PCNA were detected by western blot. (**B**) Nuclear fragmentation of LM3 and BEL-7402 cells was observed by fluorescence microscopy and Hoechst 33342 dye after treatment with MJ for 48 h (magnification 200 ×). The number of positive cells was calculated using Image-Pro Plus software 6.0 (n=6). (**C**) Apoptosis of LM3 and BEL-7402 cells was determined by flow cytometry. ATP depletion was measured by ATP detection kits. (**D**) The protein levels of Caspase-3, Caspase-9 and PARP were determined by western blot. Percent of control was recorded after Z-VAD-FMK treatment (n=3). The data are expressed as the mean±SD [*P<0.05 for MJ (1 mM) versus Control, ^#^P<0.05 for MJ (2 mM) versus MJ (1 mM), and ^^^P<0.05 for MJ (3 mM) versus MJ (2 mM)].

Sustaining proliferative signaling and resisting cell death are the most important factors in cancer cells. Therefore, we evaluated the ability of MJ to inhibit proliferation using CCK8. The HCC cell lines LM3, Huh-7, BEL-7402, Hep3B and SMMC-7721 were incubated for 6–72 h in a series of MJ concentrations (0–4 mM) and a growth curve was drawn (Figure 2A). The results showed that MJ inhibited the proliferation of hepatoma cells in a time- and dose-dependent manner. The 50 and 80 percent maximal inhibitory concentration (IC50 and IC80) at 48 h were calculated and are shown in Table 1. Proliferating cell nuclear antigen (PCNA), an indicator which reflects the state of cell proliferation as measured by western blot, also showed cell growth inhibition by MJ at 48 h (Figure 2A).

**Table 1:** Growth-inhibitory activity of MJ on HCC cells

MJ(mM)	LO2	LM3	Huh7	BEL-7402	Hep3B	SMMC-7721
**IC50**	24.30	1.88	3.71	2.54	3.45	3.29
**IC80**	158.04	5.28	13.47	8.60	10.84	9.5

Based on the synthesis results, we chose LM3 and BEL-7402 at 48h time point for subsequent experiments. Hoechst 33342 staining, flow cytometry and ATP depletion were used to assess cell death rate. Fluorescent Hoechst 33342 dye can enter normal cell membranes and cause slight staining which is increased in apoptotic cells. In addition, damaged DNA structure in apoptotic cells can combine with the dye and the P-glycoprotein pump fails to expel the dye from the cell. Various causes can lead to enhanced blue fluorescence in apoptotic cells. As shown in Figure 2B, cells in the control group showed light blue, while fluorescence intensity increased with MJ dose. Cells in the MJ (3 mM) group showed little blue color and did not display nucleus structures (P=0.001). We randomly selected six areas and counted positive cells. Significant differences (P<0.05) between the groups showed that MJ played an important role in promoting apoptosis. The results of flow cytometry (Annexin V-FITC/PI) also confirmed these findings (Figure 2C). Related protein expression (Caspase-3, Caspase-9 and PARP) indicated similar levels of apoptosis (Figure 2D). ATP is an indicator of cell metabolic activity. The bioluminescent method determines the energy from ATP using firefly luciferase to define the number of living cells. The upward curve of ATP depletion within 24 h in LM3 and BEL-7402 showed a reduced number of living cells indicating necrosis (Figure 2C). These results demonstrated that MJ induced necrosis and apoptosis of liver cancer cells.

Z-VAD-FMK, a cell-permeable, irreversible broad-spectrum caspase inhibitor, blocks all features of apoptosis. We treated cell lines LM3 and BEL-7402 with Z-VAD-FMK (50 μM) for 1 h before incubation with MJ. The downward trend in the presence of Z-VAD-FMK was consistent with the original trend which showed that MJ-induced apoptosis is not dependent on caspase enzymes (Figure 2D and Supplementary Figure 1A). This may be associated with apoptosis inducing factor (AIF) and Bcl-2 changes mediated by mitochondria.

### Methyl jasmonate decreased glycolysis level by destroying the mitochondrial membrane potential (MMP) of HCC cells

**Figure 3:**
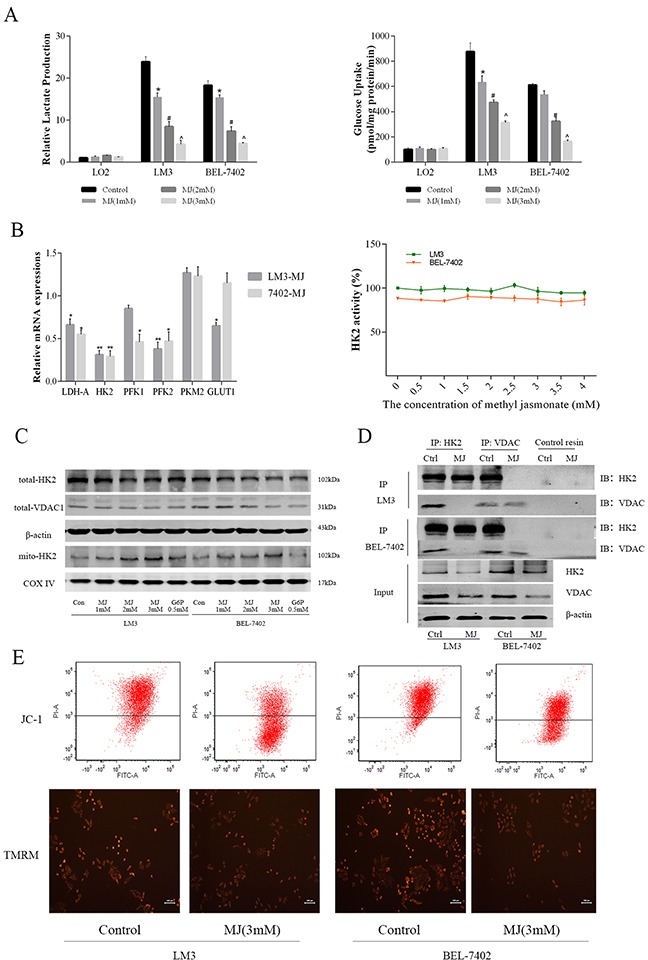
The effects of methyl jasmonate on glycolysis level in HCC cells (**A**) Normal liver cells (LO2) and HCC cells (LM3 and BEL-7402) were treated with MJ (0–3 mM) for 48 h. Lactate production and glucose uptake were analyzed and are shown as the mean±SD. [n=6, *P<0.05 for MJ (1 mM) versus Control, ^#^P<0.05 for MJ (2 mM) versus MJ (1 mM), and ^^^P<0.05 for MJ (3 mM) versus MJ (2 mM)]. (**B**) The mRNA expression of HK2, PFK, PKM, GLUT1 and LDH was determined using real-time PCR. HK2 activity in LM3 and BEL-7402 was measured after treatment with MJ for 48 h. (**C**) The protein levels of total HK2 and VDAC1, and mito-HK2 were detected by western blot. β-actin and COX IV were regarded as internal references of total and mitochondrial protein, respectively. (**D**) The expression of HK2 and VDAC1 in HCC cells was analyzed by immunoblotting (IB) after IP with HK2 and VDAC1. The input was 20 μg protein before antibody cross-linking. No signals were detected using control resin from the kit. (**E**) The MMP of LM3 and BEL-7402 with or without MJ (3 mM) was measured after JC-1 and TMRM staining. Above the line represents cells with high Δψm and below the line represents cells with low Δψm. The intensity of red fluorescence represents the amount of dye taken up by mitochondria.

It is unclear whether MJ can promote tumor cell necrosis and apoptosis through glycolysis. Normal liver cells (LO2) and two liver cancer cell lines (LM3 and BEL-7402) were treated with MJ for 24 h to evaluate lactate production and glucose uptake (Figure 3A). The results showed that MJ had almost no impact on LO2 cells, but significantly affected the energy metabolism of cancer cells in a dose-dependent manner (P<0.05). We further analyzed the gene transcription of enzymes related to glycolysis, such as HK2, LDH, PFK and GLUTs. We found differences in HK2, LDH and PFK2, with no activity change in HK2 (Figure 3B).

Glucose-6-phosphate (G-6-P), a product of HK2 catalysis, can perform feedback inhibition and was regarded as a positive control [29]. MJ treatment significantly lowered HK2 and VDAC1 expression from total protein and was similar to G-6-P at 0.5 mM at 48h, HK2 expression in the mitochondria showed the opposite trend (Figure 3C and Supplementary Figure 1B). This suggested that MJ not only inhibited transcription and the expression of HK2, but was also associated with the detachment of HK2 and VDAC1. These findings were confirmed by IP (Figure 3D). The HK2 antibody decreased VDAC1 in the control group that expressed HK2 and VDAC1, while VDAC1 disappeared after MJ treatment. The combination of HK2 and VDAC1 is closely related to mitochondrial membrane potential (MMP), thus JC-1 and TMRM were used to measure the change in MMP at 48h by flow cytometry and fluorescence microscopy imaging (Figure 3E). The results showed a reduction in potential normal cell counts after MJ treatment in LM3 and BEL-7402. In addition, strong fluorescence of TMRM indicated a decrease in normal mitochondria (Figure 3D). These results demonstrated that MJ could detach HK2 from VDAC1 in mitochondria and prevent its strong impact on glycolysis.

### Change in the expression of HK2 influenced methyl jasmonate-induced mitochondrial apoptosis in HCC cells

**Figure 4:**
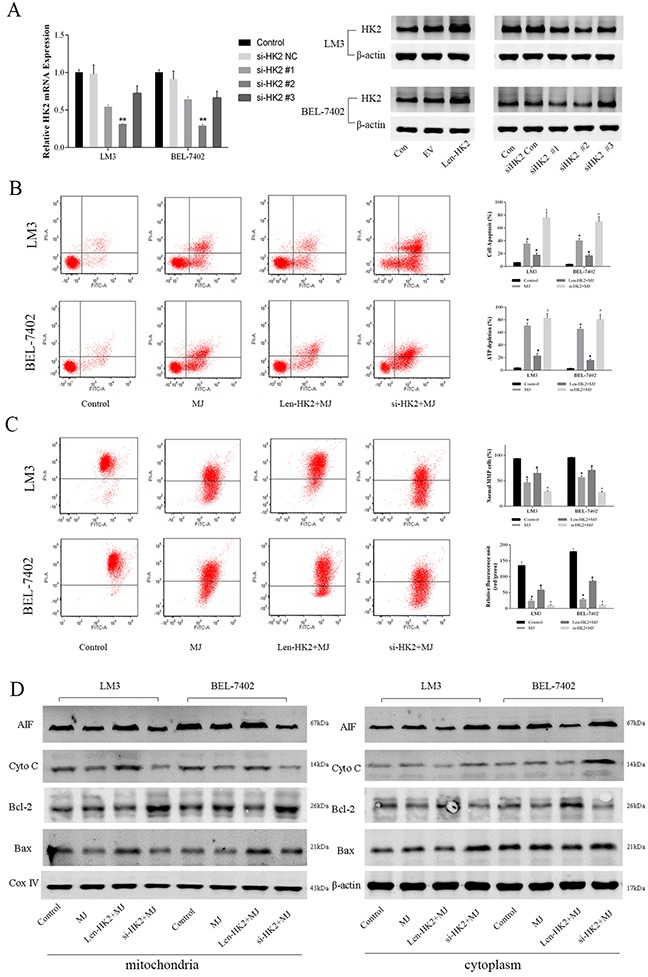
The effects of methyl jasmonate on glycolysis level following HK2 overexpression or silencing in HCC cells (**A**) The gene and protein level of HK2 in LM3 and BEL-7402 were measured after transfection by lentivirus or siRNA. The data are expressed as the mean±SD. (n=6, ^*^P<0.01 for si-HK2 #2 versus si-HK2 NC). (**B**) Apoptosis of LM3 and BEL-7402 cells was determined by flow cytometry. ATP depletion was measured by ATP detection kits. (**C**) JC-1 and TMRM staining were used to measure the MMP. Relative fluorescent units of red/green were obtained using a microplate reader. (n=3, *P<0.05 for MJ versus Control, ^#^P<0.05 for Len-HK2+MJ versus MJ, and ^^^P<0.05 for si-HK2+MJ versus MJ). (**D**) The protein expression of AIF, Cyto C, Bcl-2 and Bax in the mitochondria or cytoplasm of LM3 and BEL-7402 was measured by western blot. β-actin and Cox IV were the internal references of protein from mitochondria and the cytoplasm, respectively.

We constructed the lentivirus vector pCDH-HK2 and HK2 siRNA to up-regulate or down-regulate HK2 expression in cell lines. PCR and western blot were then performed to determine the change in expression of HK2 (Figure 4A). siRNA-HK2 #2 was then selected for subsequent experiments (P=0.011). As shown in Figure 4B, the cells with upregulated HK2 expression decreased MJ (3mM)-induced apoptosis and necrosis in LM3 ad BEL-7402 cells at 48 h (P<0.05). In contrast, the effect of MJ was improved when HK2 was downregulated in HCC cells. The changes of mitochondrial membrane potential (MMP) at 48h showed consistent trendy (Figure 4C). The results showed HK2 was the important target of MJ.

When apoptosis occurs, cyto C [30] and apoptosis inducing factor (AIF) are released from mitochondria to the cytoplasm to activate caspases and other proteins such as Bcl-2 and Bax [31]. Therefore, we measured protein expression in the mitochondria and cytoplasm of LM3 and BEL-7402, respectively (Figure 4D and Supplementary Figure 1C). The results showed that pro-apoptotic factor AIF, cyto C and Bax were reduced in mitochondria but increased in the cytoplasm after MJ treatment. When HK2 was upregulated, apoptosis was weakened, but the opposite findings were observed in the siHK2 group. As an anti-apoptotic protein, Bcl-2 was upregulated by MJ in mitochondria and showed the opposite trend to the other markers [32]. In conclusion, the effects of MJ on HCC cells were achieved by acting on HK2 which influenced the balance of the MMP.

### Methyl jasmonate enhances chemotherapeutics and the combination treatment effect in chemotherapy-resistant HCC cells

**Figure 5:**
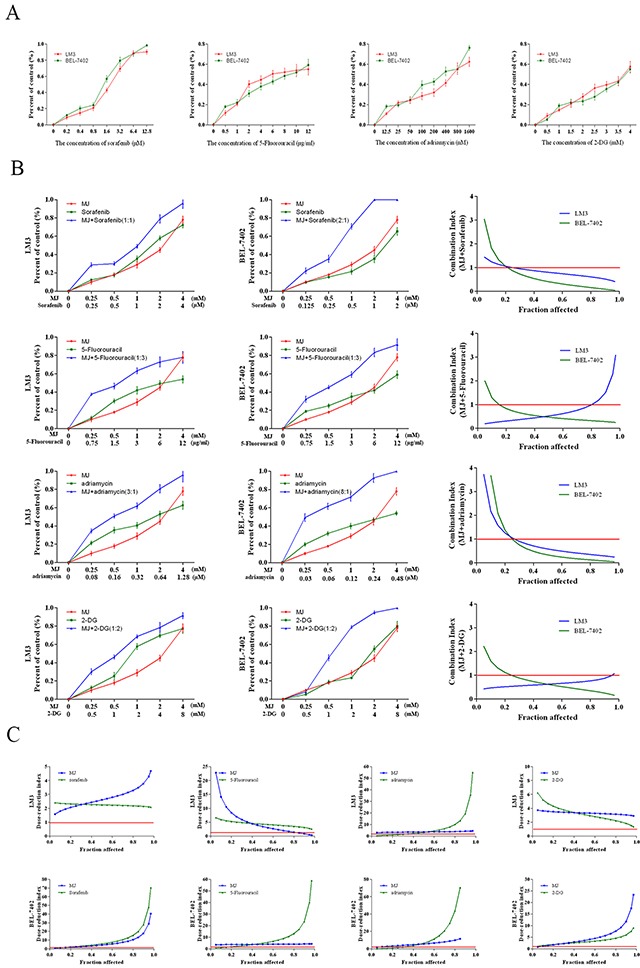
Combination treatment of MJ with sorafenib, 5-Fluorouracil, Adriamycin and 2-DG (**A**) HCC cells (LM3 and BEL-7402) were treated with sorafenib (0–12.8 μM), 5-Fu (0–12 μg/mL), adriamycin (0–1.6 μM) and 2-DG (0–4 mM) for 48 h. (**B**) HCC cells were treated with these chemotherapeutics combined with MJ for 48 h. CI values were calculated at each Fa. (**C**) DRI was analyzed for the three drugs in HCC cells at each Fa. The CCK8 kit was used to monitor cell proliferation (n=3).

Numerous clinical studies have proved that patients diagnosed with liver cancer showed no survival benefit due to chemotherapy resistance. While the emergence of targeted drugs, such as sorafenib has improved survival, this drug is not widely used due to its high price, serious side effects combined with the high resistance of some tumor cells. Therefore, it is very important to improve sensitivity to chemotherapeutic drugs and reduce dosage. For these purposes, HCC cells LM3 and BEL-7402 were treated with sorafenib (0–12.8 μM, IC50 of 1.78 and 1.28 μM), 5-Fu (0–12 μg/mL, IC50 of 6.71 and 8.41 μg/mL), adriamycin (0–1.6 μM, IC50 of 0.6 and 0.3 μM) and 2-DG (0–4 mM, IC50 of 3.9 and 4.3 mM) for 48 h (Figure 5A). 2-DG, which was previously reported to have a combination effect, was regarded as a positive control [33]. The fraction affected (Fa) values (indicating the fraction of cells inhibited after drug exposure) were then obtained after MJ was combined with a series of chemotherapeutic drugs at different concentrations (Figure 5B). The dose was a fixed drug ratio of 1:1 at the IC50. The CI (combination index) and DRI (dose reduction index) values were calculated for each Fa (Figure 5B & 5C). As expected, the results demonstrated synergism (CI<1) at a limited Fa range, while combinations resulted in a favorable dose reduction (DRI>1) of the three drugs (Figure 5C). The DRI-Fa plots showed that the chemotherapeutic doses of sorafenib, 5-Fu and adriamycin were significantly reduced and were similar to the proved effect of 2-DG.

### Methyl jasmonate increased sorafenib-induced HCC cell growth inhibition *in vivo*

**Figure 6:**
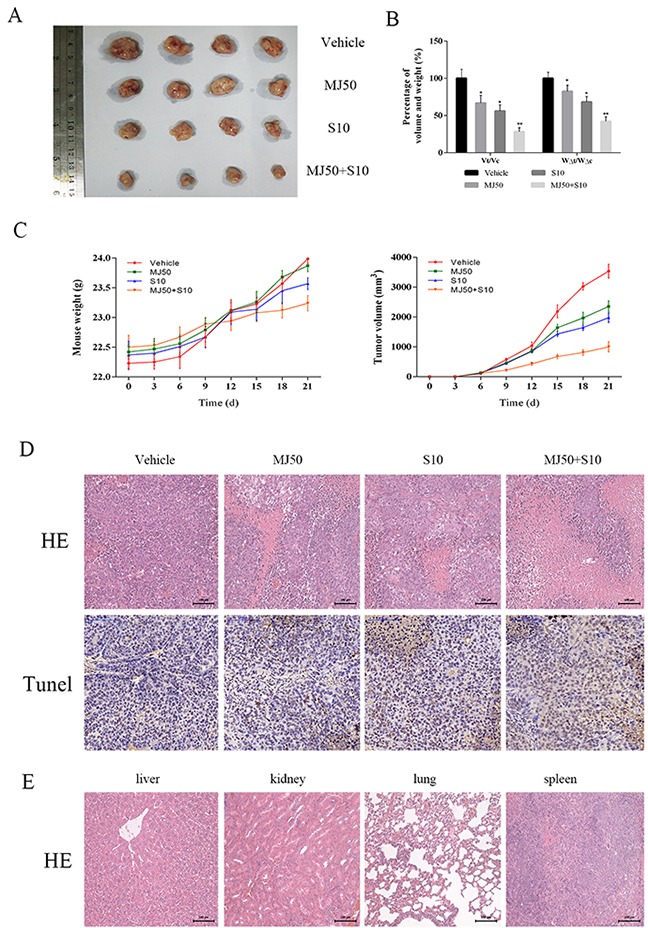
The effects of methyl jasmonate combined with sorafenib *in vivo* (**A**) Gross observation of HCC-LM3 cell xenograft tumors in nude mice. (**B**) The changes in tumor volume and mouse weight are expressed as the mean±SD. (n=6, *P<0.05 for MJ50 versus Vehicle, #P<0.05 for S10 versus Vehicle, and ^P<0.05 for MJ50+S10 versus MJ50 or S10). (**C**) The changes in tumor volume and body weight were recorded at the time points indicated. (**D**) HE and TUNEL staining of tumors show the level of necrosis and apoptosis. The number of cells with positive TUNEL staining was calculated using Image-Pro Plus software 6.0 (n=6). (**E**) HE staining of liver, kidney, lung and spleen showed no significant changes (magnification 200 ×).

We have verified growth inhibition of HCC cells by MJ and its combination with other targeted and chemotherapeutic drugs. These results prove that MJ can partly increase the sensitivity of chemotherapeutic drugs at a certain concentration range. In order to further verify these effects, we constructed a mouse xenograft model using HCC-LM3 cells for *in vivo* testing. Nude mice received a daily dose of saline, MJ (50 mg/kg), sorafenib (10 mg/kg) and sorafenib (10 mg/kg) combined with MJ (50 mg/kg) by gavage after the formation of neoplasia (diameter 0.5 cm). After 21 days, a significant change was observed with the naked eye (Figure 6A). According to statistical analysis, the increase in tumor volume and mouse weight in the MJ group and sorafenib group were significantly retarded. The combination group (P=0.001, 0.005, respectively) showed remarkable development (Figure 6B). Mouse weight and tumor volume in the vehicle group increased rapidly but were slower in the other groups, as shown in Figure 6C. HE and TUNEL staining indicated the level of necrosis and apoptosis, respectively. Nuclear fragmentation in the combination group suggested a strong degree of necrosis consistent with apoptosis and corresponded with the number of brown particles (Figure 6D). Importantly, MJ had no effect on the liver, kidney, lung and spleen (Figure 6E). Taken together, these findings indicate that MJ enhanced the inhibition of sorafenib-induced cell growth and when combined with sorafenib, necrosis and apoptosis were promoted in HCC cells.

## DISCUSSION

Tumor growth is highly dependent on glycolysis, therefore, inhibitors including glycolytic enzymes and regulators of metabolism targeting glycolysis can effectively inhibit cell proliferation [5, 34]. Hexokinase (HK) is the first key enzyme of glycolysis, and HK2 with high specific expression is negatively related to programmed cell death [35]. We determined the gene expression and products of glycolysis in normal liver cells and HCC cells from several perspectives. The results showed that the gene transcription of HK2 was significantly higher and the copy number was more than three times greater in HCC cells compared with LO2 cells. This was most obvious in the LM3 cell line with high invasiveness. In addition, the consistency of gene and protein expression in HK2 may be due to post-transcriptional regulation as well as post-translational regulation. Furthermore, the degradation of mRNA and protein and the modified folding may lead to differences in the abundance and protein expression [36, 37]. Accordingly, lactate and glucose consumption also increased with reduced OXPHOS protein expression. These findings showed that glycolysis was dominant in malignant tumors.

Firstly, we found that MJ had a significant inhibitory effect on the growth of HCC cells, but had little effect on normal liver cells. Tumor cells maintained high ATP/ADP as well as NADH/NAD^+^ ratios, and after MJ treatment, increased ATP depletion was associated with greater necrotic death in cells [38]. Markers of apoptosis, caspases and PARP were used to assess apoptosis, which was found to be independent of caspases. Therefore, we suspected that the mechanism may be associated with a difference in energy metabolism between normal cells and cancer cells. Secondly, we further examined the relationship between MJ and glycolysis. A significant reduction in lactate production and glucose uptake occurred in HCC cells following MJ treatment with no obvious changes in normal liver cells. The gene expression screening results showed a close relationship with glycolysis, and HK2 was the most significant gene. Interestingly, MJ did not change HK2 activity. G-6-P, an HK2 inhibitor was used as a positive control to define the action of MJ. The crosscurrent in separated mitochondrial proteins confirmed that HK2 may be shifted without ontology change. The results of IP demonstrated that the invalidation of HK2 was attributed to its dissociation with the voltage-dependent anion channel (VDAC).

**Figure 7:**
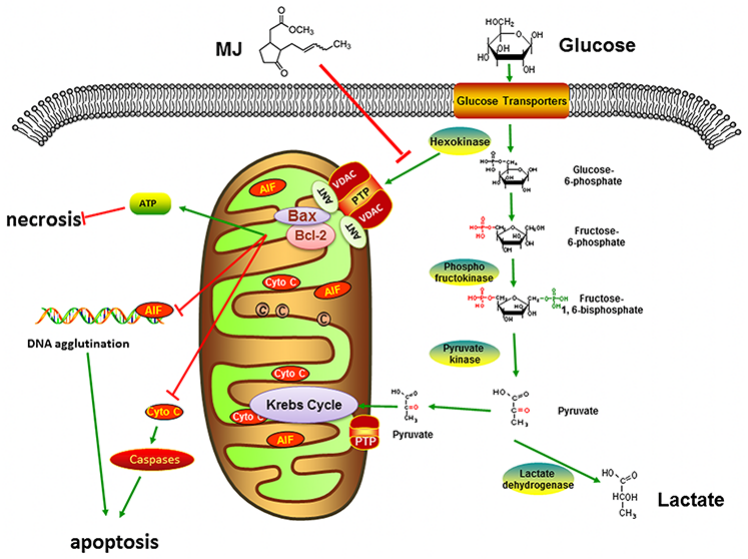
Mechanism of MJ action MJ damaged the HK2/VDAC1 complex to open the PTP leading to increased mitochondrial membrane permeability. Blocked ATP synthesis caused by mitochondria injury hampered OXPHOS and led to cell necrosis. The mitochondrial membrane potential was instantly reduced to release cytochrome C and AIF into the cytoplasm, respectively indicated typical intrinsic and caspases-independent apoptotic pathways with Bcl-2 and Bax changes and nuclear DNA agglutination.

Mitochondria are of vital importance in tumor energy metabolism [39, 40]. The permeability transition pore (PTP) which runs through the outer and inner membrane located on the mitochondrion surface, consists of VDAC, adenine nucleotide translocase (ANT) and cyclophilin D [41, 42]. HK2 can combine with VDAC1 through the hydrophobic N-terminal region to use ATP generated by mitochondria to promote glycolysis [43]. MJ damaged the HK2/VDAC1 complex to open the PTP leading to increased mitochondrial membrane permeability. The mitochondrial membrane potential (MMP, ΔΨm) was instantly reduced to release cytochrome C into the cytoplasm, similar to the typical intrinsic apoptotic pathways with Bcl-2 and Bax changes [44, 45]. On the other hand, damaged mitochondrial function caused AIF transfer from mitochondria to the cytoplasm causing nuclear DNA agglutination. Unlike traditional apoptosis, the pathway was not inhibited by broad spectrum caspase inhibitors. Blocked ATP synthesis caused by mitochondria injury hampered OXPHOS and led to cell necrosis [46]. When the HK2 gene was up-regulated artificially, the effect of MJ was reversed and the down-regulated HK2 gene was synergistic with MJ. This ensured that MJ dissociated the combination of HK2 and VDAC1 to block energy for tumor growth by inhibiting glycolysis (Figure 7).

Most liver cancers are inoperable and are treated with chemotherapy. However, liver cancer has extremely low susceptibility to chemotherapy which may be related to multi-drug resistance genes [14, 47]. Furthermore, most chemotherapeutic agents have significant side effects due to necessary high doses. Targeted drugs, such as sorafenib have a satisfactory effect but are expensive. The known glycolysis inhibitor, 2-DG had a favorable combination effect with MJ and could be regarded as a positive control [33]. When MJ was combined with sorafenib, adriamycin and 5- Fu, the same growth inhibitory rate was achieved, but the dosage was significantly reduced. Thus, MJ could be used as a chemosensitizer and either used alone or combined with other agents due to its rapid synthesis and low cost. *In vivo*, MJ alone showed a favorable anti-tumor effect, but showed greater inhibition of cancer growth when combined with sorafenib. In addition, toxicology experiments demonstrated that MJ did not cause obvious pathological damage to liver, lung, spleen and kidney and is safe and reliable.

In conclusion, we found that the major effect of methyl jasmonate in HCC cells was profoundly different to its effect on normal cells: methyl jasmonate interfered with the growth of cancer cells by inhibiting glycolysis. The underlying mechanism was dependent on necrosis and apoptosis by detaching HK2 from VDAC1 causing loss of mitochondrial function. The favorable effect of methyl jasmonate could be improved and then used in future clinical treatment, especially in chemotherapy-resistant patients.

## MATERIALS AND METHODS

### Cell culture and reagents

The HCC cell lines LM3, Huh-7, BEL-7402, Hep3B, SMMC-7721 and normal liver cells LO2 were purchased from the Chinese Academy of Sciences Committee Type Culture Collection Cell Bank (Shanghai, China). The LO2 cells were cultured in RPMI-1640 with 10% fetal bovine serum, 100 U/mL penicillin, and 100 μg/mL streptomycin, and the HCC cells lines were cultured in high glucose Dulbecco's modified Eagle's medium (DMEM, Logan, UT, USA) at 37°C in a humidified atmosphere of 5% CO_2_.

MJ, 5-Fu, adriamycin, 2-DG, dimethyl sulfoxide (DMSO) and glucose-6-phosphate (G-6-P) were purchased from Sigma Aldrich (St. Louis, MO, USA) and stored at −20°C for future use. Z-VAD-FMK and sorafenib were obtained from Selleck Chemicals (Shanghai, China). The final concentrations of DMSO did not exceed 0.1%. All antibodies were purchased from Cell Signaling Technology (Danvers, MA, USA). The Annexin V/PI apoptosis detection kit was purchased from BD Biosciences (San Jose, CA, USA), the co-immunoprecipitation (IP) kit was from Thermo Fisher Scientific (Waltham, MA, USA) and the antibodies (HK2 and VDAC1) used in IP were from Proteintech Group (Chicago, IL, USA).

### Cell proliferation and combination analysis

The cell lines were incubated for 6–72 h with a series of drug treatments after being plated at a density of 2 × 10^4^ cells/mL in 96-well plates (100 μL medium per well). The Cell Counting kit (Dojindo Laboratories, Tokyo, Japan) was used at a ratio of 1 to 100 and cell viability was measured using a microplate reader (Synergy H4, BioTek, Winooski, VT, USA) at a wavelength of 450nm. Cell inhibition rate (%) =1-[ (OD_drug_ –OD_blank_)/(OD_control_ –OD_blank_)]. The IC50, combination index (CI) and dose reduction index (DRI) values were calculated using Compusyn software (Paramus, NJ, USA). The CI was calculated by: CI = (D)1/(Dx)1+(D)2/(Dx)2+(D)1(D)2/(Dx)1(Dx)2, in which (Dx)1 and (Dx)2 are the doses of drug 1 and drug 2 alone, and (D1) is the dose of drug 1 in combination, and (D2) the dose of drug 2 in combination. Where CI = 1 indicates that the two drugs have additive effects, CI <1 indicates more than additive effects (“synergism”) and CI>1 indicates less than additive effects (“antagonism”). This was designated as the DRI: (DRI)1 = (Dx)1/(D)1 and (DRI)2 = (Dx)2/(D)2. DRI>1 showed that combinations could result in reduced drug doses compared with the doses for each drug alone.

### Glucose uptake, lactate production and O_2_ consumption

The cells were cultured in uptake buffer (140 mM NaCl, 2 mM KCl, 1 mM KH2PO4, 10 mM MgCl2, 1 mM CaCl2, 5 mM glucose, 5 mM L-alanine, 5 mM indomethacin, and 10 mM HEPES/Tris, pH 7.4) containing 1 μCi/mL 2-DG at 37°C for 30 min after being washed twice. The radioactivity of the cells was then counted using a liquid scintillation counter when solubilized in 0.1% sodium dodecyl sulfate (SDS). Quantification of protein was performed to normalize the above results. Lactate was measured using the microplate reader according to the manufacturer's protocol. With regard to O_2_ consumption, 1 × 10^7^ cells/mL were collected and then tested using a 110 Fiber optic oxygen monitor (Instech, Plymouth Meeting, PA, USA) and is expressed as nmol O_2_/million cells/min.

### Reverse transcription (RT)-polymerase chain reaction and quantitative real-time (qRT) PCR

Total RNA was extracted by Trizol, chloroform and isopropyl alcohol and then dissolved in diethyl pyrocarbonate (DEPC) water. After concentration determination, approximately 2000 ng RNA were transcribed into cDNA using the reverse transcription kit (TaKaRa Biotechnology, Dalian, China). A 7900HT fast real-time PCR system (Applied Biosystems, Foster City, CA, USA) was used to determine the gene expression level of HK2, PFK and LDH according to the manufacturer's protocol. Primers used in the experiment are shown in Table 2.

**Table 2:** Nucleotide sequences of primers used for qRT-PCR

Gene		Primer sequence (5′—3′)
HK2	Forward	GAGCCACCACTCACCCTACT
	Reverse	CCAGGCATTCGGCAATGTG
LDH	Forward	AGGTGCGAACCTCCTGATG
	Reverse	CGGTGCCGAATGGGATGAT
PFK1	Forward	GCTGGGCGGCACTATCATT
	Reverse	TCAGGTGCGAGTAGGTCCG
PKM2	Forward	ATGTCGAAGCCCCATAGTGAA
	Reverse	TGGGTGGTGAATCAATGTCCA
GLUT1	Forward	GCCAGAAGGAGTCAGGTTCAA
	Reverse	TCCTCGGAAAGGAGTTAGATCC
GAPDH	Forward	TGTGGGCATCAATGGATTTGG
	Reverse	ACACCATGTATTCCGGGTCAAT

### Mitochondrial protein extraction and western blot analysis

The mitochondrial fractions were separated and purified from the collected HCC cells using a Mitochondrial Isolation Kit. Total protein was extracted using radio immunoprecipitation assay (RIPA) buffer containing protease inhibitor. The bicinchoninic acid (BCA) protein assay (Thermo Fisher Scientific) was used to determine the concentration of the prepared protein.

Before protein analysis, all samples were mixed with 5× sodium dodecyl sulfate-polyacrylamide gel electrophoresis (SDS-PAGE) sample loading buffer and boiled for 10 min. After separation by SDS-polyacrylamide gels, the bands were excised and transferred onto polyvinylidene fluoride (PVDF) membranes which were blocked for 60 min with 5% bovine serum albumin (BSA) dissolved in PBS. The blots were then incubated overnight at 4°C with the corresponding antibody: PCNA (1:1000), HK2 (1:500), PFK (1:1000), OXPHOS (1:100), Caspase-3 (1:500), Caspase-9 (1:500), PARP (1:1000), VDAC1 (1:1000), AIF (1:500), Cyto C (1:1000), Bcl-2 (1:1000), Bax (1:500), Cox IV (1:2000), and β-actin (1:2000). After washing three times with PBS containing 0.1% Tween 20 (PBST), the secondary antibody (anti-rabbit or anti-mouse IgG (1:2000) was added and incubated for 1 h at room temperature. The Odyssey two-color infrared laser imaging system (LI-COR Biosciences, Lincoln, NE, USA) was used to scan the membranes.

### Hoechst 33342 staining

HCC cell lines LM3 and BEL-7402 were collected at a density of 1 × 10^6^ cells/mL after MJ treatment. Hoechst 33342 dye dissolved in PBS (1:200) was added to the fixed cells for 20 min in the dark. The images were obtained by fluorescence microcopy (Leica, Wetzlar, Germany).

### Flow cytometry-based Annexin-V/PI assay

HCC cells in logarithmic growth phase were seeded into six-well plates and treated as indicated. After 48 h, the cells were digested by trypsin and centrifuged after being washed with PBS. The sediments were collected, suspended in 1× binding buffer and incubated for 20 min at room temperature with Annexin-V/PI (BD Biosciences). The samples were examined by flow cytometry (Cytomics FC500; Beckman Coulter, Fullerton, CA, USA).

### ATP depletion

The ATP Bioluminescence Assay Kit (Roche Applied Science, Mannheim, Germany) was used to quantify intra-cellular ATP according to a standard protocol. Briefly, the cells were mixed with 50 μL of Luciferase Reagent after being lysed with Cell Lysis Reagent on ice. The ATP concentration was calculated and normalized to protein levels.

### HK2 activity assay

HK2 activity was measured using the Hexokinase Colorimetric Assay Kit (Biovision, Milpitas, CA, USA). Approximately 1×10^6^ cells were incubated with 200 μL HK Assay Buffer for 10 min on ice and centrifuged at 12,000 g for 5 min. A 1-50 μL sample (40 μg) was added to each well, and the final volume was adjusted to 50 μL with HK Assay Buffer. A parallel sample well was prepared as a background control to avoid interference due to NADH in the sample. After mixing sufficient reagents for the number of assays, the samples were incubated for 20–60 min at room temperature and measured at OD450 nm. Hexokinase activity (mU/mL) = NADH amount/(reaction time × sample volume) × dilution factor.

### Co-Immunoprecipitation (Co-IP)

The Thermo Scientific Pierce Co-Immunoprecipitation kit (Thermo Fisher Scientific) was used to detect the isolation of native protein complexes. According to the manufacturer's protocol, the amino link plus coupling resin and Pierce control agarose resin were used for antibody crosslinking. The final elution buffer was mixed with the lane marker sample buffer and heated at 100°C for 5 min before applying the SDS gels. A total of 10 μg antibodies (HK2 and VDAC1) were added to the antibody mixture and stored for regeneration. Traditional Co-IP methods using Protein A or G result in co-elution of antibody heavy and light chains which may co-migrate with relevant bands, masking important results. However, the kit includes optimized buffers for protein binding which avoids extraneous factors.

### Mitochondrial membrane potential detection

5,5′,6,6′-tetrachloro-1,1′,3,3′-tetraethylbenzimidazolcarbocyanine (JC-1) and tetramethylrhodamine methyl ester (TMRM) are fluorescent probes used to measure mitochondrial membrane potential (MMP, ΔΨm). Red fluorescence shows normal MMP with relatively high ΔΨm, while green fluorescence indicates damaged mitochondrial function with decreased ΔΨm. A suspension of HCC cells in logarithmic growth phase was prepared and inoculated into six-well plates. Following MJ treatment for 48 h, the cells were incubated with 0.1 μg/mL of JC-1 or TMRM at 37°C for 20 min. The cells were then washed with dyeing buffer twice for flow cytometry detection or observation by fluorescence microscopy. The remaining suspension was tested to obtain relative fluorescent units (RFU) of red (590 nm) or green (530 nm).

### Plasmid construction, lentivirus packaging and infection

A full-length cDNA encoding the HK2 sequence which was amplified from 293T cDNA was cloned into the EcoR I/BamH I sites in the pCDH-CMV-MCSEF1-GFP vector (System Biosciences, Mountain View, CA, USA). Lipofectamine™2000 (Invitrogen, Carlsbad, CA, USA) was used for co-transfection of pCDH-CMVMCS-EF1-GFP empty vector or pCDH-HK2 in 293T cells to confirm the plasmid sequences. The LM3 and BEL-7402 cell lines were then infected overnight in the presence of 8 μg/mL polybrene (Sigma-Aldrich). The empty vector was regarded as the control. The transduction efficiency was measured by PCR and western blotting.

### siRNA transfection

HCC cells at a density of 2 × 10^5^/well were inoculated into six-well plates and incubated for 24 h. Serum-free Opti-MEM was used to wash the cells twice and Lipofectamine™2000 combined with 50 μM siRNA were added to the cell culture for transfection (8 h). The HCC cells were then treated with MJ with a corresponding cell culture for 48 h and both PCR and western blotting were performed to analyze the transfection efficiency. The sequences used in the experiment are shown in Table 3.

**Table 3:** Nucleotide sequences of siRNA-HK2

Si-RNA		Sequence (5′—3′)
Si-HK2 #1	Forward	GCUCCGAAAUGUGAUGUGU
	Reverse	ACACAUCACAUUUCGGAGC
Si-HK2 #2	Forward	GAGGAACAAAUUUCCGGGU
	Reverse	ACCCGGAAAUUUGUUCCUC
Si-HK2 #3	Forward	GAGAAUCAGAUCUAUGCCA
	Reverse	UGGCAUAGAUCUGAUUCUC

### Animal experiments and ethics statement

A total of 16 nude mice were maintained under conditions of 22°C, 55% humidity and a 12 h light/12 h dark cycle, and monitored every day. All animal experiments were performed according to the National Institutes of Health Guidelines for the Care and Use of Laboratory Animals and were approved by the Animal Care and Use Committee of Shanghai Tongji University, China. LM3 HCC cells were subcutaneously injected into the upper flank region of all mice at a density of 5×10^6^ (100 μL). After one week, 1.0 mm^3^ subcutaneous tumor tissue was placed in the infection area. All mice were randomly divided into four groups (four in each group) as follows:

Group I, vehicle: saline

Group II, MJ50: 50 mg/(kg·d) MJ

Group III, S10: 10 mg/(kg·d) sorafenib

Group IV, MJ50+S10: 50 mg/(kg·d) MJ and 10 mg/(kg·d) sorafenib

All drugs were dissolved or suspended in saline containing 1% DMSO and administered orally for 21 days. Tumor volume and mouse weight were measured every 5 days and the mice were subsequently killed by cervical dislocation after anesthesia. Tumor tissue, liver, kidney, spleen and lung were removed and imaged using a high-definition digital camera.

### Hematoxylin and eosin (H&E) staining

A portion of tumor tissue was fixed with 4% paraformaldehyde and then dehydrated. The samples were then embedded in paraffin to prevent crystallization. Sections 5 μm thick were cut and stored at room temperature. Hematoxylin and eosin were added to stain the nuclear region and cytoplasm, respectively. Histopathology changes were observed under a light microscope.

### Terminal deoxynucleotidyl transferase (TdT)-mediated dUTP nick end labeling (TUNEL) staining

The paraffin sections were dehydrated with ethanol after being dewaxed twice in xylene for 5–10 min and incubated in Proteinase K without DNase at a concentration of 20 μg/mL for 15–30 min. Reaction buffer was dropped into the sections and observed by light microscopy according to the manufacturer's protocol. Apoptotic cells were observed to have a dark brown color.

### Statistical analysis

All data are expressed as the mean ± standard deviation (SD) and statistical analysis was performed using SPSS 20.0 software (IBM, Chicago, IL, USA). Differences between groups in PCR, CCK8 assay, flow cytometric analysis, tumor volume and weight analysis were determined by one-way analysis of variance (ANOVA) using the Student-Newman-Keuls (SNK) method. In all cases, P<0.05 was considered statistically significant.

## SUPPLEMENTARY MATERIALS FIGURES


