# Supplementary material for: Methyl jasmonate leads to necrosis and apoptosis in hepatocellular carcinoma cells via inhibition of glycolysis and represses tumor growth in mice
Source: Oncotarget. 2017 Apr 27;8(28):45965–80. doi: 10.18632/oncotarget.17469 (PMC5542241; doi:10.18632/oncotarget.17469)
Supplement: Supplementary file 1 [file oncotarget-08-45965-s001.pdf]

Methyl jasmonate leads to necrosis and apoptosis in hepatocellular carcinoma cells via inhibition of glycolysis and represses tumor growth in mice

SUPPLEMENTARY MATERIALS

SUPPLEMENTARY FIGURE

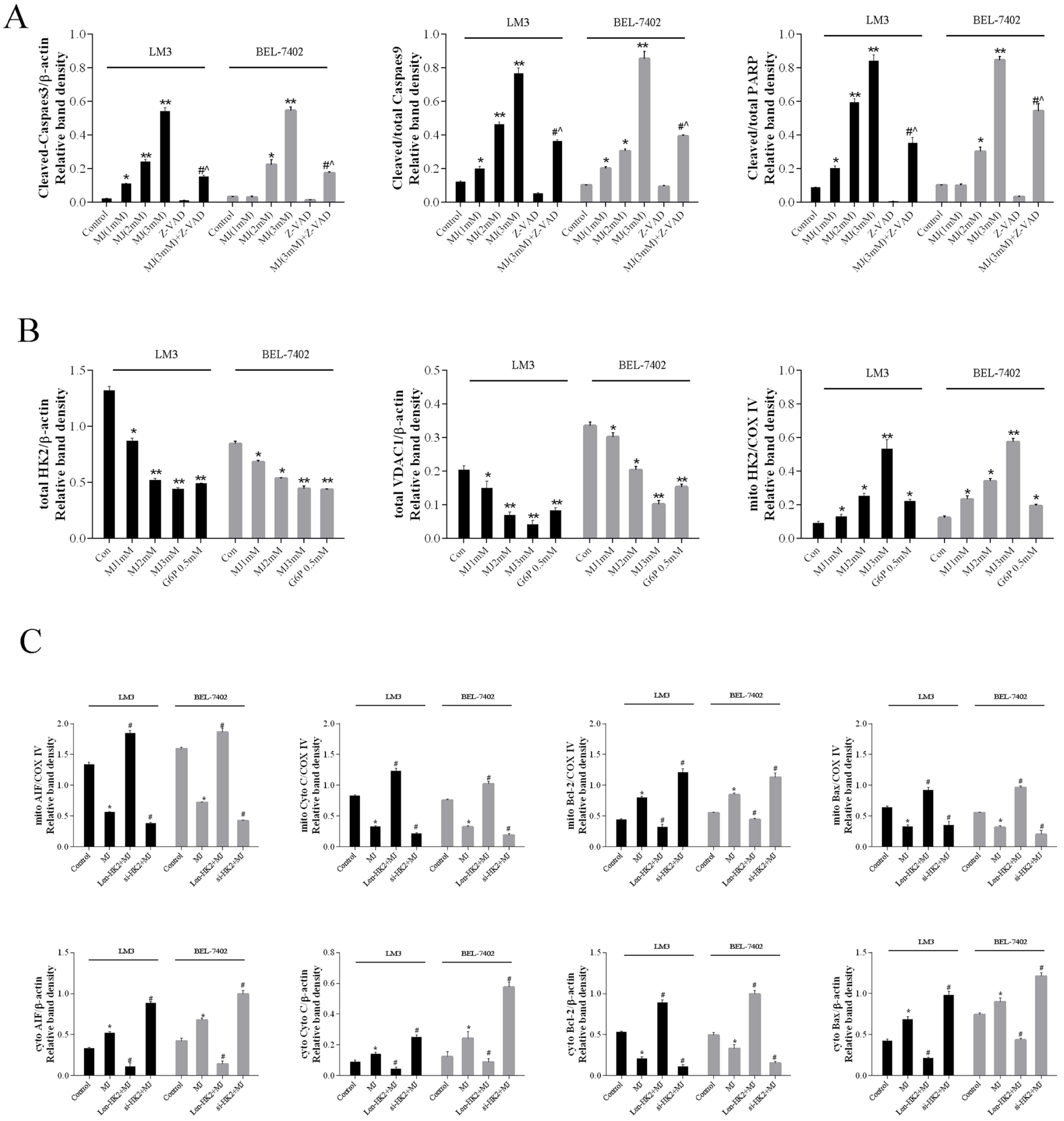

Supplementary Figure 1: The relative band density of western blot images. (**A**) The relative band density of cleaved caspase-3, caspase-9 and PARP. The data are expressed as the mean±SD [*P<0.05 and **P<0.01 for MJ versus Control, #P<0.05 for MJ (3 mM)+Z-VAD versus Z-VAD, and ^P<0.05 for MJ (3 mM)+Z-VAD versus MJ (3 mM)]. (**B**) The relative band density of HK2 and VDAC1. [*P<0.05 and **P<0.01 for MJ and G6P versus Con]. (**C**) The relative band density of related apoptotic proteins. [*P<0.05 for MJ versus Control, #P<0.05 for Len(si)-HK2+MJ versus MJ].
